# Preliminary assessment of automated radiology report generation with generative pre-trained transformers: comparing results to radiologist-generated reports

**DOI:** 10.1007/s11604-023-01487-y

**Published:** 2023-09-15

**Authors:** Takeshi Nakaura, Naofumi Yoshida, Naoki Kobayashi, Kaori Shiraishi, Yasunori Nagayama, Hiroyuki Uetani, Masafumi Kidoh, Masamichi Hokamura, Yoshinori Funama, Toshinori Hirai

**Affiliations:** 1 Department of Diagnostic Radiology, Graduate School of Medical Sciences, Kumamoto University, 1-1-1 Honjo, Chuo-ku, Kumamoto-shi, Kumamoto, 860-8556 Japan; 2 Department of Medical Physics, Faculty of Life Sciences, Kumamoto University, Honjo 1-1-1, Kumamoto, 860-8556 Japan

**Keywords:** Radiology report, Computed tomography, Deep learning, Large language model, Generative pre-trained transformer

## Abstract

**Purpose:**

In this preliminary study, we aimed to evaluate the potential of the generative pre-trained transformer (GPT) series for generating radiology reports from concise imaging findings and compare its performance with radiologist-generated reports.

**Methods:**

This retrospective study involved 28 patients who underwent computed tomography (CT) scans and had a diagnosed disease with typical imaging findings. Radiology reports were generated using GPT-2, GPT-3.5, and GPT-4 based on the patient’s age, gender, disease site, and imaging findings. We calculated the top-1, top-5 accuracy, and mean average precision (MAP) of differential diagnoses for GPT-2, GPT-3.5, GPT-4, and radiologists. Two board-certified radiologists evaluated the grammar and readability, image findings, impression, differential diagnosis, and overall quality of all reports using a 4-point scale.

**Results:**

Top-1 and Top-5 accuracies for the different diagnoses were highest for radiologists, followed by GPT-4, GPT-3.5, and GPT-2, in that order (Top-1: 1.00, 0.54, 0.54, and 0.21, respectively; Top-5: 1.00, 0.96, 0.89, and 0.54, respectively). There were no significant differences in qualitative scores about grammar and readability, image findings, and overall quality between radiologists and GPT-3.5 or GPT-4 (*p* > 0.05). However, qualitative scores of the GPT series in impression and differential diagnosis scores were significantly lower than those of radiologists (*p* < 0.05).

**Conclusions:**

Our preliminary study suggests that GPT-3.5 and GPT-4 have the possibility to generate radiology reports with high readability and reasonable image findings from very short keywords; however, concerns persist regarding the accuracy of impressions and differential diagnoses, thereby requiring verification by radiologists.

**Supplementary Information:**

The online version contains supplementary material available at 10.1007/s11604-023-01487-y.

## Introduction

In recent years, significant advancements have been made in the field of diagnostic imaging due to the development of computed tomography (CT), magnetic resonance imaging (MRI), and other imaging equipment, as well as advances in scanning methods. As a result, the utilization of these imaging modalities has been on the rise in many countries. The role of radiology reports has become extremely important in the diagnosis and treatment of various diseases [1]. However, the increase in reading and reporting work has led to the problem of radiologist burnout [2]. Radiologists are facing an overwhelming amount of data to analyze and report, which can lead to errors and delays in making radiology reports. Therefore, it is crucial to find ways to address the issue of radiologist burnout while maintaining the quality of radiology reports.

The rapid development of artificial intelligence (AI) has demonstrated its value across various fields, and it has become a prominent topic in the realm of diagnostic radiology [3–7]. In radiology reports, AI has shown promise in the interpretation of simple chest radiographs [8, 9], but its performance in more complex modalities like CT and MRI remains limited. The generative pre-trained transformer (GPT), an advanced large language model (LLM) [10], has recently gained significant attention due to its ability to produce human-like sentences with ChatGPT, a chat system based on GPT-3.5. Additionally, recent research has shown that GPT performed at or near the passing threshold for all three exams of the USMLE without any specialized training or reinforcement [11], and the usefulness for the transformation and the summarization of radiology reports [12, 13]. On the other hand, it has been reported that LLMs such as the GPT series have the potential to generate inaccurate content, referred to as hallucinations [14]. Consequently, we hypothesize that GPT models have the potential for generating radiology reports or assisting radiologists in writing such reports; however, research exploring these specific applications remains limited.

In this preliminary study, we embarked on an initial evaluation of the GPT series’ potential to generate radiology reports from simple imaging findings, comparing its performance to that of radiologist-generated reports.

## Methods

### Study design and population

This retrospective study was approved by the institutional review board. Informed consent for this retrospective study was waived by the institutional ethics committee. This single-center, retrospective study was conducted to evaluate the potential of the GPT series in generating radiology reports and compare their performance with radiologist-generated reports. For the purpose of calculating the Mean Average Precision (MAP) in this study, a list of image findings and their corresponding differential diagnoses were required to feed into the GPT series. Hence, we distilled a concise list of 28 image findings along with their associated differential diagnoses from the “Radiology Review Manual, 8th ed” (https://www.wolterskluwer.com/en/solutions/ovid/radiology-review-manual-3568), a reference extensively used in radiology, as presented in Table 1. The 28 image findings selected for this study are commonplace in radiology reports, and it has been confirmed by the authors, as per the review manual, that differential diagnoses can be reasonably inferred solely from these image findings. Subsequently, a search was performed within the radiology reporting system, focusing on reports containing these basic findings within the designated timeframe of January 2020 to February 2023. The portions of the radiologists’ reports corresponding to “image findings”, “impression”, and “differential diagnosis” of the selected patients were utilized as benchmarks for comparison against the reports generated by the GPT series.

**Table 1:** Findings and final diagnoses

Organ	Finding (*)	Final diagnosis
Brain	1. Calcified intracranial mass	1. Anaplastic oligodendroglioma
	2. Cyst with mural nodule	2. Pilocytic astrocytoma
	3. Dense cerebral mass	3. Medulloblastoma
	4. Deep ring-enhancing lesion	4. Metastatic brain tumor
	5. Well-defined superficial enhancing mass	5. Meningioma
	6. Multifocal enhancing lesions	6. Malignant lymphoma
	7. Cystic mass in cerebellar hemisphere	7. Hemangioblastoma
	8. Enhancing supra- and intrasellar mass	8. Pituitary adenoma
	9. Suprasellar mass with calcification	9. Craniopharyngioma
Lung	10. Random nodules	10. Multiple pulmonary metastases
	11. Centrilobular nodules without ground-glass opacities	11. Atypical mycobacterial infection
	12. Pulmonary mass with air bronchogram	12. Lung cancer
	13. Multiple pulmonary calcifications	13. Old tuberculosis
Mediastinum	14. Fat-containing mediastinal mass left atrial mass	14. Lipoma
	15. Right atrial mass	15. Myxoma
	16. Left atrial mass	16. Thrombus
Liver	17. Ring-enhancing targetlike liver mass	17. Intrahepatic cholangiocarcinoma
	18. Hypervascular mass in normal liver	18. Hepatic adenoma
	19. Hypervascular mass in chronic liver disease	19. Hepatocellular carcinoma
	20. Hypervascular mass with central scar	20. Focal nodular hyperplasia
	21. Delayed phase-enhancing lesion	21. Hemangioma
Pancreas	22. Hypervascular pancreatic tumors	22. Islet cell tumor
	23. Pancreatic cyst with solid component	23. Mucinous cystadenocarcinoma
	24. Macrocystic lesion of pancreas	24. Intraductal papillary mucinous neoplasm
Others	25. Mucocele of appendix	25. Appendiceal mucinous neoplasm
	26. Heterogeneous fat-containing retroperitoneal mass	26. Liposarcoma
	27. Fat-containing adrenal mass	27. Adrenal adenoma
	28. Fat-containing renal mass	28. Angiomyolipoma

*The findings are as stated in the “Radiology Review Manual, 8th ed.” and the capitalization and other formatting adhere to that source

We identified 28 patients whose radiology reports included selected basic findings as shown in Table 1 between October 2002 and February 2023, and these individuals were the subjects of this investigation. Subsequently, we included 28 patients (12 males and 16 females) aged 5 to 86 years, with a mean age of 59.0 ± 24.1 years. We searched the patients’ medical records and confirmed their final diagnoses, which were determined by surgical procedures, biopsy, or follow-up observations, and documented these in Table 1. All assessments were conducted successfully for these patients.

### Radiology report generation

**Fig. 1:**
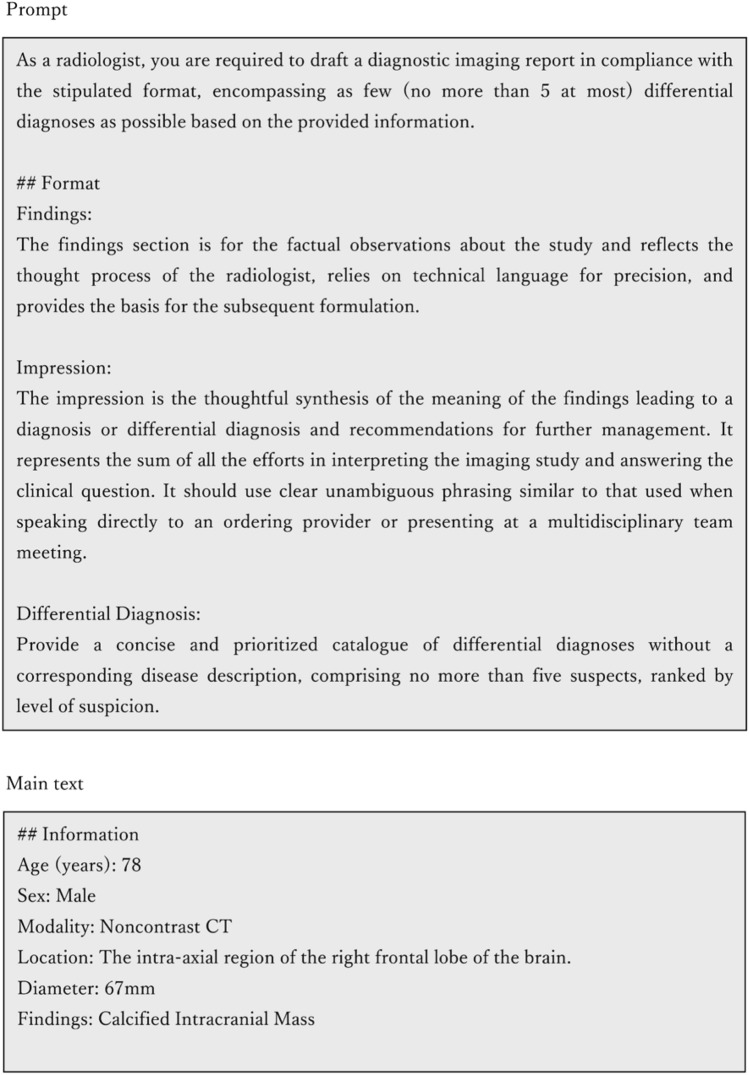
An example of “Prompt” and “Information of a patient”. Before inputting actual patient data into the GPT series, it is necessary to provide guidance on the role and type of text to be generated. This instruction is called a “Prompt”. The prompt serves as a way to inform the language model about the context and desired output. In this case, the example prompt explains that the output should be from the perspective of a radiologist and the purpose of each part of the radiology report. The “Prompt” is common for all patients, and only the “Main text” portion varies for each individual patient. This approach ensures that the language model receives consistent contextual information while tailoring the generated report to the specific details of each patient's case

**Fig. 2:**
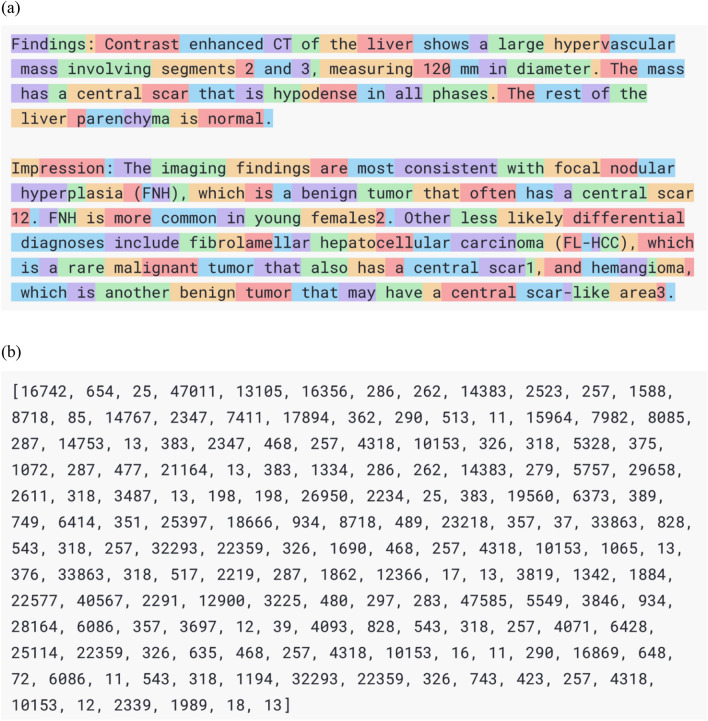
A visualization of tokens used in generative pre-trained transformer (GPT) series. GPT series and other transformer-based models perform language processing (**a**) in units called “tokens”, each of which has a unique identifier (**b**). The task of text generation is internally processed as selecting the token with the highest probability of appearing after a particular sequence of tokens. This approach allows the model to generate coherent and contextually appropriate text by predicting and selecting the most likely tokens to follow a given input sequence

Radiology reports were generated using GPT-2, GPT-3.5, and GPT-4 models based on the patient’s age, gender, disease site, and imaging findings. To generate radiology reports using the GPT series, we used the customized prompt before the above information of each patient (Fig. 1). This prompt was generated based on a previously reported radiology reporting guide [1]. To access GPT-2 and GPT-3.5, the researchers utilized OpenAI’s application programming interface (API), which can be found at https://openai.com/. This API is a system that processes user inputs automatically on the service provider’s end and subsequently returns the processed data. In this case, the system was employed to send prompts and patient information to OpenAI, which then generated the radiology reports. During the period of this study, the model equivalent to GPT-2 was “text-davinci-002”, and the one corresponding to GPT-3.5 was “gpt-3.5-turbo”; both models were used in this study. Unfortunately, in the study period, GPT-4’s API had not yet been officially released, and the permission for use by the public was limited to a customized version for Microsoft Bing Chat. As a result, the researchers input the above prompt and information into Microsoft Bing Chat to obtain the radiology reports. The GPT series utilized in this paper is an advanced form of the transformer, undergoing a conversion process termed “token”. Details about this process can be found in the supplementary material. Figure 2 illustrates an example of a radiology report with visualized tokens generated using the “tokenizer” (https://platform.openai.com/tokenizer).

For the sake of comparison, previously generated radiologist-authored reports written in Japanese from the same patients were also included in the study. Any unstructured sections or content written in Japanese was translated into English by a board-certified radiologist with over 20 years of research experience in the English language, and another board-certified radiologist with 10 years of English research experience checked translated English reports. The translations were based on the previously reported Radiology Reporting Guide [1] as well as reports generated by GPT series and followed the categories “Findings”, “Impressions”, and “Differential Diagnosis”. We used these translated reports as the radiology reports written by radiologists for comparison with radiology reports generated by GPT series.

### Quantitative analysis of differential diagnosis

The quality of different diagnoses in radiology reports was assessed quantitatively by calculating the top-1, top-5 accuracy, and MAP of radiology reports for radiologists, GPT-2, GPT-3.5, and GPT-4. Top-1 and top-5 accuracies were defined as the proportion of cases where the final diagnosis was ranked first and within the top five, respectively. The MAP was calculated to provide a comprehensive evaluation of the ranking of correct diagnoses by comparing the generated differential diagnoses to the reference differential diagnoses in the “Radiology Review Manual, 8th ed”. Precision was calculated for each relevant diagnosis at each rank (i.e., if the correct diagnosis appeared at rank 1, 2, 3, etc.), and the average of these precision values was computed to obtain the MAP. This measure accounts for both the precision and the recall of the generated differential diagnoses, offering a more holistic assessment of the radiology report quality.

### Qualitative analysis

One board-certified radiologist and one radiology resident, blinded to the report’s origin, were tasked with independently evaluating multiple aspects of the radiology reports. These aspects included grammar and readability, image findings, impression, differential diagnosis, and overall quality. To facilitate a systematic and objective evaluation, a 4-point Likert scale was employed, with the following rating categories: 1 (poor), 2 (fair), 3 (good), and 4 (excellent).

For the assessment of grammar and readability, the evaluators considered the clarity and coherence of the report, the accuracy of syntax, and the appropriateness of the language used. In evaluating imaging findings, we assessed whether imaging findings that corresponded to the abnormal findings entered were appropriately described and whether contradictory findings were written. In assessing impressions, the evaluator considered whether the considerations drawn from the imaging findings were consistent and reasonable to guide an accurate differential diagnosis. In evaluating differential diagnoses, the validity, and scope of the alternative diagnoses listed in the report were reviewed based on the findings entered, age, and sex. Lastly, the overall quality of the reports was evaluated based on the integration of the aforementioned criteria. Disagreements between the two evaluators were resolved through discussion until a consensus was reached.

### Statistical analysis

We compared the accuracy of differential diagnoses between radiologists and the GPT series using McNemar’s test. Qualitative results are presented as the median and interquartile range (IQR) due to the non-normal distribution of the data, and differences between the scores of radiologists and those of the GPT series were compared using the Wilcoxon signed-rank test. For multiple comparisons, Holm’s correction was applied. To assess inter-reader agreement, weighted Cohen’s Kappa analyses were conducted (kappa ≤ 0.40, poor agreement; 0.40 < kappa ≤ 0.60, moderate agreement; 0.60 < kappa ≤ 0.80, good agreement; and kappa > 0.80, excellent agreement). All statistical analyses were performed using the free programming software Python version 3.8.5 (https://www.python.org). A two-tailed *p*-value < 0.05 was deemed significant.

## Results

Table 2 shows the results of the quantitative analysis of the different diagnoses. Top-1 and Top-5 accuracies for the different diagnoses were highest for radiologists, followed by GPT-4, GPT-3.5, and GPT-2, in that order (Top-1: 1.00, 0.54, 0.54, and 0.21, respectively; Top-5: 1.00, 0.96, 0.89, and 0.54, respectively). In Top-1 accuracies, there were significant differences between the radiologists and GPT series (*p* < 0.01). In Top-5 accuracies, there was a significant difference between the radiologists and GPT-2 (*p* < 0.05); however, there were no significant differences between the radiologists and GPT-3.5 (*p* = 0.50) and GPT-4 (*p* = 1.00). MAPs for the different diagnoses were highest for radiologists, followed by GPT-4, GPT-3.5, and GPT-2, in that order (0.97, 0.26, 0.45, and 0.54, respectively).

**Table 2:** Quantitative analysis

	Radiologists	GPT-2	GPT-3.5	GPT-4 (Bing)	*p*-value^1^		
					Radiologists vs. GPT-2	Radiologists vs. GPT-3.5	Radiologists vs. GPT-4
Top-1 accuracy	1.00	0.21	0.54	0.54	< 0.001***	0.002**	0.002**
Top-5 accuracy	1.00	0.46	0.89	0.96	< 0.001***	0.50	1.00
MAP	0.97	0.26	0.45	0.54			

*GPT* Generative pre-trained transformer, *MAP* mean average precision

^1^**p < 0.01; ***p < 0.001

**Fig. 3:**
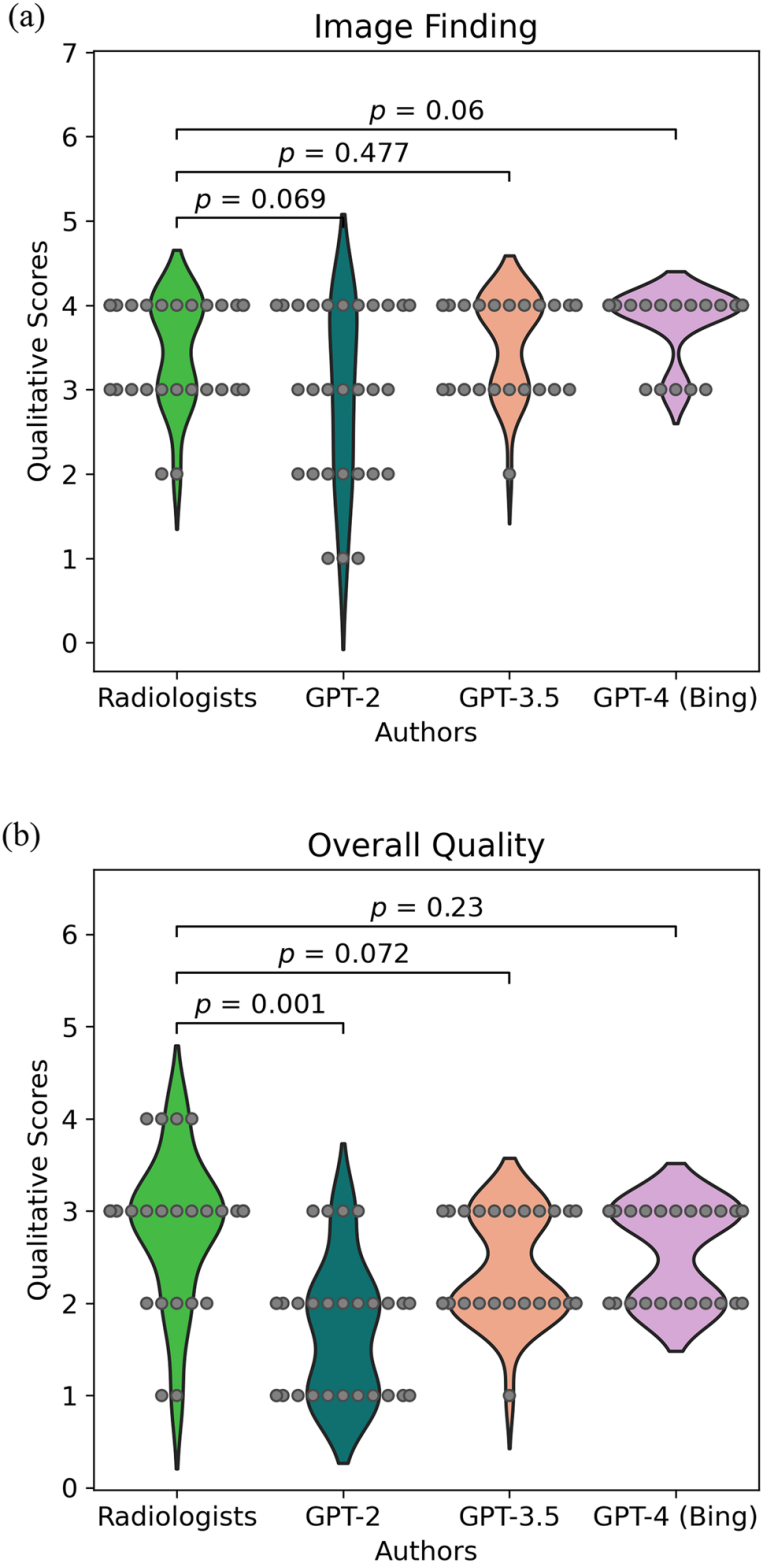
Qualitative analysis. Violin plots show qualitative analysis of the image findings (**a**) and the overall quality (**b**)

Table 3 and Fig. 3 show the results of the qualitative analysis of the radiology reports. There were no significant differences in qualitative scores about grammar and readability, image findings, and overall quality between radiologists and GPT-3.5 or GPT-4 (*p* > 0.05). However, qualitative scores of radiologists for impression and differential diagnosis were significantly higher than those of the GPT series (*p* < 0.05). The Kappa analysis indicated poor to moderate concordance (grammar and readability: *κ* = 0.72, image findings: *κ* = 0.59, impression: *κ* = 0.46, differential diagnosis: *κ* = 0.41, overall quality: *κ* = 0.42).

**Table 3:** Qualitative analysis

	Radiologists^1^	GPT-2^1^	GPT-3.5^1^	GPT-4 (Bing)^1^	*p*-value^2^		
					Radiologistss vs. GPT-2	Radiologists vs. GPT-3.5	Radiologists vs. GPT-4
Grammar & readability	4.0 (3.0, 4.0)	3.0 (3.0, 3.25)	4.0 (4.0, 4.0)	4.0 (4.0, 4.0)	0.02*	0.29	0.29
Image finding	4.0 (3.0, 4.0)	3.0 (2.0, 4.0)	4.0 (3.0, 4.0)	4.0 (4.0, 4.0)	0.069	0.477	0.060
Impression	3.0 (3.0, 4.0)	2.0 (1.0, 2.25)	2.5 (2.0, 3.0)	3.0 (2.0, 3.0)	< 0.001***	0.002**	0.003**
Differential diagnosis	3.0 (3.0, 3.0)	2.0 (2.0, 2.0)	2.0 (2.0, 3.0)	2.0 (2.0, 3.0)	< 0.001***	0.002**	0.034
Overall quality	3.0 (2.75, 3.0)	2.0 (1.0, 2.0)	2.0 (2.0, 3.0)	3.0 (2.0, 3.0)	0.001**	0.072	0.23

*GPT *Generative pre-trained transformer, *MAP* mean average precision

^1^Median (interquartile range)

^2^*p < 0.05; **p < 0.01; ***p < 0.001

**Fig. 4:**
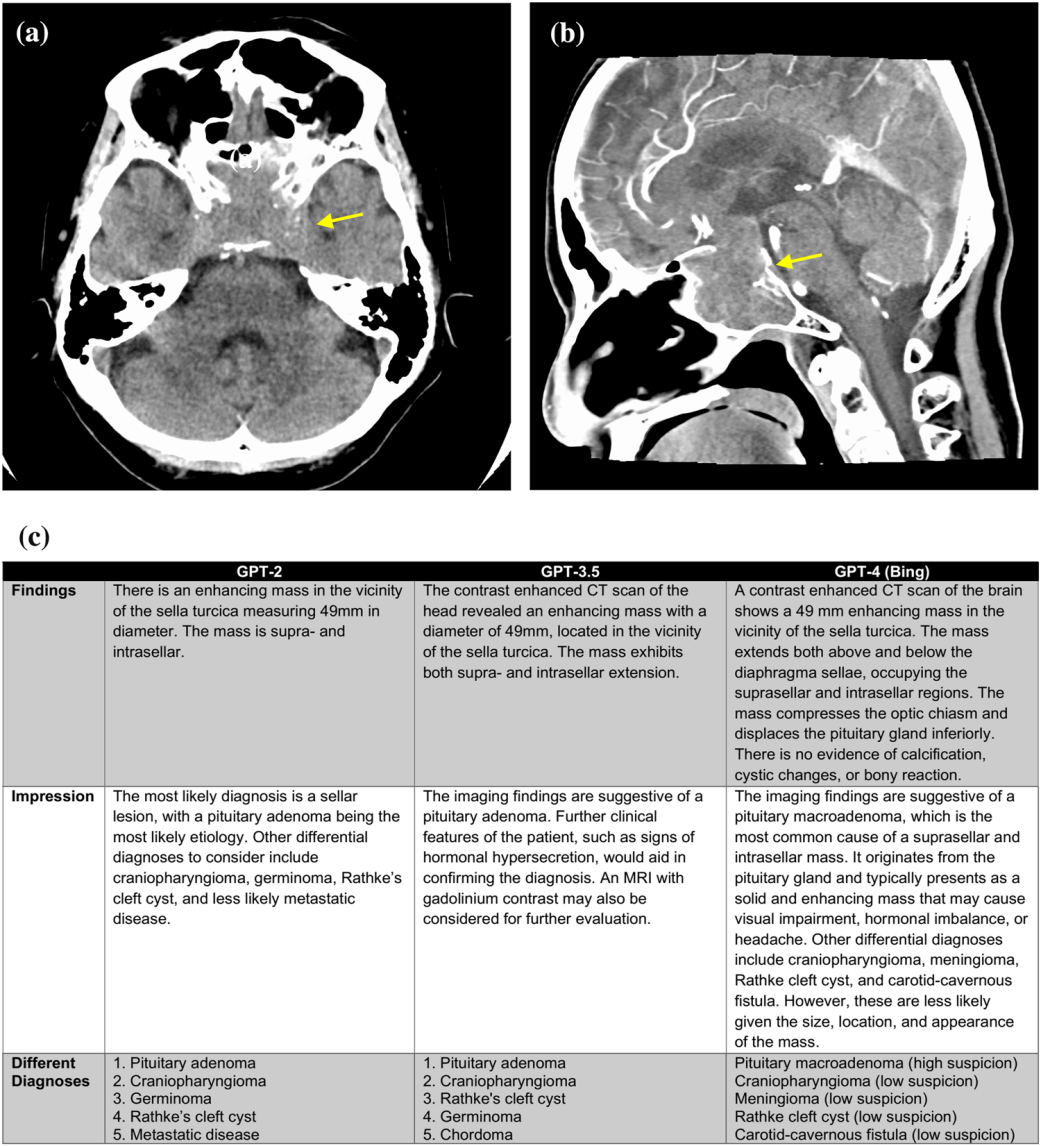
A 69-year-old female patient with a suspected pituitary adenoma. Non-contrast CT axial image (**a**), contrast-enhanced CT sagittal image (**b**) and generated radiology reports by GPT series (**c**) are shown. A tumor with homogeneous enhancement is observed from the sella turcica to the suprasellar region, suggesting a pituitary adenoma. Information input other than the prompt is “Age (years): 69, sex: female, modality: contrast enhanced CT, location: the vicinity of the sella turcica, diameter: 49 mm, findings: enhancing supra- and intrasellar mass”. The GPT-2 report is a simple report written according to the input information, and the differential diagnosis seems relatively reasonable. In the GPT-3.5 report, both the findings and impression sections are more detailed than in the GPT-2 report. The GPT-4.0 report is overall quite similar to a human-generated report, and the differential diagnosis is reasonable. However, it includes information that was not input, such as calcification and cystic degeneration

**Fig. 5:**
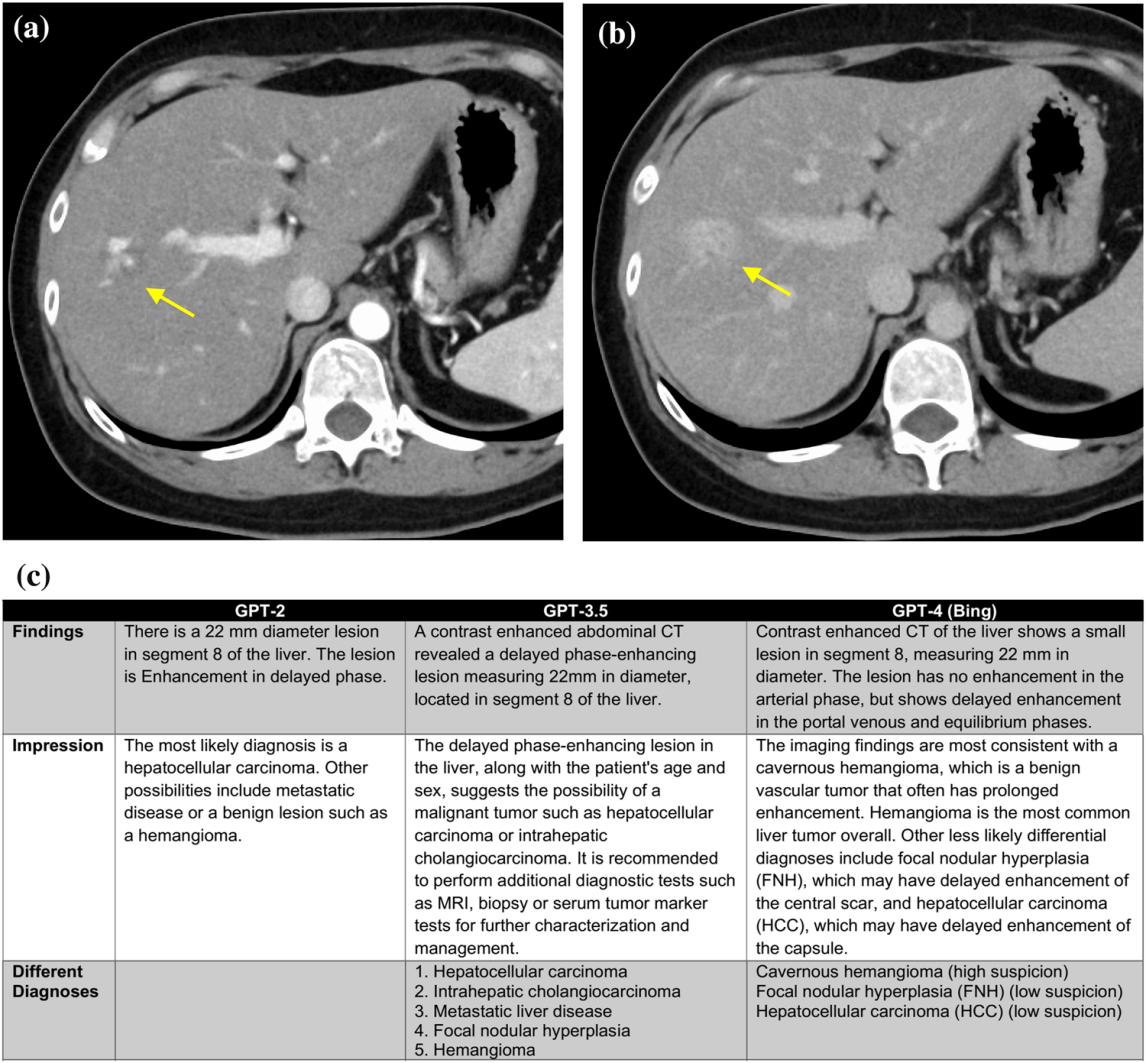
A 31-year-old female with a hepatic hemangioma. The contrast-enhanced CT arterial phase (**a**) shows heterogeneous enhancement within the lesion, and the venous phase (**b**) reveals a generally stronger enhancement than the liver parenchyma, consistent with typical findings of a hemangioma. Generated radiology reports by GPT series (**c**) are also shown. The information inputted besides the prompt is “Age (years): 31; sex: female; modality: contrast enhanced CT; location: segment 8 in the liver; diameter: 22 mm; findings: delayed phase-enhancing lesion”. In the GPT-2 generated report, a list of differential diagnoses is not even created, and the impression primarily suspects hepatocellular carcinoma. In the GPT-3.5 generated report, although the format is well organized, hepatocellular carcinoma is still listed as the top differential diagnosis. The GPT-4.0 generated report is generally quite good, with reasonable differential diagnoses

**Fig. 6:**
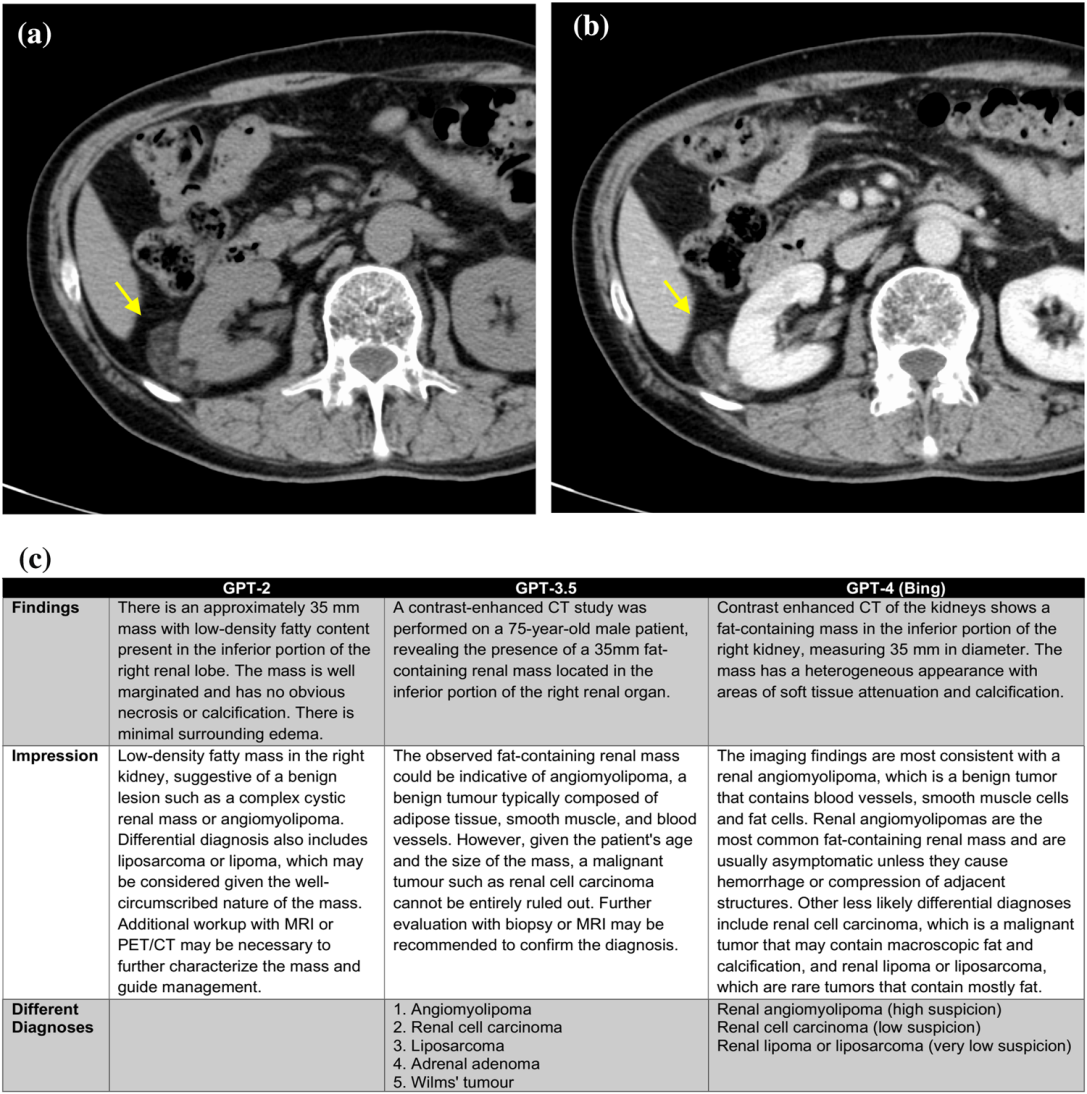
A 75-year-old male with an angiomyolipoma in the right kidney. A non-contrast CT (**a**) and contrast-enhanced CT (**b**) reveal a fatty renal mass in the right kidney. Generated radiology reports by GPT series (**c**) are also shown. The information inputted besides the prompt is “Age (years): 75; sex: male; modality: contrast-enhanced CT; location: the inferior portion of the right kidney; diameter: 35 mm; findings: fat-containing renal mass”. In the GPT-2 generated report, the possibility of a renal tumor accompanied by surrounding edema is low, and a list of differential diagnoses is not even created. In the GPT-3.5 generated report, although the lesion is located in the kidney, the differential diagnoses include adrenal adenoma. In the GPT-4.0 generated report, the overall quality is quite good; however, there is a description of “calcification” in the image findings, which was not part of the input information

Representative cases and reports produced by GPT series are shown in Figs. 4, 5 and 6.

## Discussion

The present study evaluated the feasibility of employing the GPT series (GPT-2, GPT-3.5, and GPT-4) to generate radiology reports based on concise imaging findings in comparison to those generated by radiologists. Our findings reveal that, although GPT-3.5 and GPT-4 are commendable in their ability to generate readable and appropriate “image findings” and “Top-5 differential diagnoses” from very limited information, they fall short in the accuracy of impressions and differential diagnoses even for very basic and representative findings of CT. Consequently, these results underscore the continued importance of radiologist involvement in the validation and interpretation of radiology reports generated by GPT models.

GPT is a pre-trained language model that uses a transformer architecture [15] for natural language processing. It was first introduced by OpenAI in 2018 with the release of GPT-1, which was trained on a large corpus of text data using unsupervised learning techniques and had 117 million parameters [10]. GPT-2 with 1.5 billion parameters was released in 2019 and was notable for its ability to generate coherent and realistic text [16]. In 2020, OpenAI released GPT-3 with 175 billion parameters, which was even more powerful and capable of performing a wide range of language tasks [17]. ChatGPT is a specific implementation of the GPT model that is designed for conversational applications using GPT-3.5, which employs a similar architecture to the original GPT-3 but is fine-tuned using reinforcement learning from human feedback [18]. While details about GPT-4 have not been fully disclosed at the time of this study, it is said to be a more natural and accurate natural language model than GPT-3.5. However, the ability of the GPT series to generate radiology reports has not been fully evaluated.

The preferred results of this study are that GPT-3.5 and GPT-4 have the potential to produce radiology reports with human-like readability and grammar from simple imaging findings, and the Top-5 differential diagnoses generated by GPT-3.5 and GPT-4 also demonstrated human-like abilities. This is a noteworthy finding, as it highlights the ability of these advanced language models to synthesize complex and coherent reports from minimal input data, which could facilitate faster and more efficient radiology reporting. In addition, although it may be slightly different from the original usage of LLM, the results of our study suggest GPT3.5 and GPT-4 have internal medical knowledge reserves about diagnostic radiology, and it has a possibility of the models’ capacity to provide a comprehensive and relevant list of potential diagnoses that can aid in clinical decision-making and improve patient care.

On the other hand, it is essential to recognize that the accuracy of Top-1 differential diagnosis, MAP, and the qualitative score of the impression and different diagnoses of the GPT-generated reports are inferior to those of radiologist-generated reports. The result, in which the differential diagnosis from a radiologist’s report—derived from a more comprehensive set of information—is superior to a GPT-generated report based solely on age, gender, location, and rudimentary imaging findings, is indeed expected. However, it is clear from the qualitative analysis that GPT-4 can generate reports similar to those of radiologists, and it would be quite difficult for a physician ordering a CT scan to differentiate them based solely on the generated reports. The results of this study suggest that LLM, which can generate practical “image findings”, “impression”, and “differential diagnosis” with only very simple keywords, is likely to be very useful as an aid in creating diagnostic imaging reports. However, LLM should be used only as an aid, and differential diagnosis should be made by a radiologist who can make a comprehensive judgment.

Another important potential limitation of the LLM, including the GPT series, is the possibility that these models may generate findings with content that cannot be directly linked to the input information, a phenomenon known as ‘hallucination” [19]. LLMs fundamentally select the next most probable word without necessarily considering logical connections or coherence. Consequently, they may generate content unrelated to the input data but highly associated with the generated text. The mechanism of hallucinations has not been fully elucidated yet [19], and preventing hallucinations can be difficult even in language models designed to mitigate this issue, with reports indicating that hallucinations may sometimes be amplified instead [20]. This issue was observed in the current study, suggesting that caution is needed when applying LLMs to medical reports. Additionally, the evaluators had differing opinions on whether to consider the output of the GPT as detailed or as a hallucination, which resulted in poor to moderate agreements in qualitative analysis. One evaluator may have appreciated the additional details provided by the GPT, believing that they could potentially enhance the radiology report. On the other hand, other evaluators might have viewed the extra details as hallucinations, as they were not directly related to the input information and could lead to inaccuracies or confusion in the diagnostic process. As such, it might be crucial to develop strategies for mitigating these hallucinations and ensuring the accuracy of the generated radiology reports. In the future, incorporating domain-specific knowledge into the training of GPT models may allow them to generate more contextually accurate reports. However, confirmation by a radiologist would be essential to ensure the clinical utility and safety of the radiology reports generated by GPT.

There are limitations in our investigation. Firstly, a limitation of this preliminary study is the relatively small sample size of 28 patients with simple image findings. In clinical settings, amalgamating multiple findings is important; however, the chosen image findings in this study were grounded in the authors’ judgment, solely referencing one textbook. Such a methodology can induce selection bias, potentially constraining the applicability of the outcomes. Therefore, additional investigations with a larger and more diverse patient population, as well as more complex image findings, are necessary to corroborate and expand upon these initial results. Secondly, this study was conducted at a single center and by non-native English speakers, potentially introducing biases related to specific institutional practices and the ability of English reading or writing of the radiologists involved. Thirdly, LLMs, including the GPT series, are still in development, and in fact, GPT-4 has become available for use via API before submission. Additionally, GPT-4 (Microsoft Bing), used in this study, has the advantage of being able to search for the latest information on the internet, which potentially makes it more advantageous compared to other models. Moreover, various GPT models are being refined. The results of this study pertain to the time of journal submission and may change in the future. Fourth, the potential for hallucination, a notable weakness inherent to LLMs, could not be specifically assessed in this study. This is due to the evaluators not being given the patient’s history to prevent biased assessments of the report quality. As such, unless the evaluator has a comprehensive understanding of the patient’s background, differentiating between detailed reporting and hallucination could prove difficult in such applications. Lastly, the retrospective design of the study could have led to various biases. Furthermore, with this study design, we cannot evaluate the impact of GPT-generated reports on the radiologists’ efforts. Future research involving larger, multicenter, prospective studies with diverse patient populations and a focus on evaluating the impact on radiologists’ efforts is warranted to validate and expand upon these findings.

In conclusion, our preliminary study suggests that GPT-3.5 and GPT-4 have the possibility to generate radiology reports with high readability and reasonable image findings from very short keywords; however, concerns persist regarding the accuracy of impressions and differential diagnoses, thereby requiring verification by radiologists.

### Supplementary Information

Below is the link to the electronic supplementary material.

Supplementary file1 (DOCX 20 KB)

## Data Availability

The datasets generated or analyzed during the study are available from the corresponding author on reasonable request.

## Abbreviations

AI: Artificial intelligence
CT: Computed tomography
GPT: Generative pre-trained transformer
LLM: Large language model
MAP: Mean average precision
MRI: Magnetic resonance imaging
USMLE: United States medical licensing examination
