# Supplementary material for: Preliminary assessment of automated radiology report generation with generative pre-trained transformers: comparing results to radiologist-generated reports
Source: Jpn J Radiol. 2023 Sep 15;42(2):190–200. doi: 10.1007/s11604-023-01487-y (PMC10811038; doi:10.1007/s11604-023-01487-y)
Supplement: Supplementary file 1 — Supplementary file1 (DOCX 20 KB) [file 11604_2023_1487_MOESM1_ESM.docx]

The transformer - base architecture of the GPT series - is a unique neural network architecture that has revolutionized natural language processing by eschewing the use of convolutional filters and hidden states, which are commonly found in traditional Convolutional Neural Networks and Recurrent Neural Networks [1]. Instead, transformers employ a structure known as "tokens" to represent input data. These tokens serve as the building blocks for creating contextualized word embeddings that effectively capture the semantic relationships between words in each sequence. Another key innovation in transformers is the integration of an attention mechanism, a scoring system that dynamically weighs the importance of different tokens based on their contextual relevance. This attention mechanism enables transformers to efficiently capture long-range dependencies and contextual information, resulting in superior performance across a range of NLP tasks. Moreover, transformers are designed to predict the continuity of tokens within a sequence, allowing them to generate coherent and contextually appropriate text. In this study, the GPT series creates a series of sentences by successively predicting words with a high likelihood of being used in radiology reports and the words that follow them based on the input data. Figure 2 shows an example of a radiology report with visualized tokens created using “tokenizer” (<https://platform.openai.com/tokenizer>).

1. Vaswani A, Shazeer N, Parmar N, Uszkoreit J, Jones L, Gomez AN, et al. Attention Is All You Need [Internet]. arXiv; 2017 [cited 2023 Feb 27]. Available from: http://arxiv.org/abs/1706.03762
